# Management of Rare Case of Missed Fracture‐Dislocation of Second and Third CMC Joints: A Missed Injury Case Report

**DOI:** 10.1002/ccr3.70239

**Published:** 2025-02-18

**Authors:** Yeshi Dorji, Ugyen Thrinley

**Affiliations:** ^1^ Department of Orthopedic, Military Hospital Army Medical Service Thimphu Bhutan; ^2^ Department of Orthopedic Jigme Dorji Wangchuck National Referral Hospital Thimphu Bhutan

**Keywords:** carpometacarpal dislocation, isolated, misdiagnosis, open reduction, rare

## Abstract

Isolated fracture‐dislocation of the second and third carpometacarpal joints (CMC) is very rare, and it is commonly misdiagnosed. Injury to this joint is usually associated with high‐energy trauma, like a motor vehicle accident. Despite correct diagnosis, optimal treatment for these injuries is still controversial. We present a 31‐year‐old male patient with a history of one‐month‐old injury to his right hand following a motorcycle accident. Initially, he was treated by one of the orthopedic surgeons elsewhere and misdiagnosed as having blunt trauma to the hand. On presentation, he complained of persistent pain and swelling with deformity of the right hand following the initial treatment he received. Apparently, only AP and Oblique radiographs of the hand were taken. Treatment On clinico‐radiological evaluation, he was found to have a fracture dislocation of 2nd and 3rd CMC joints. He was managed with open reduction and percutaneous pinning with K‐wires. Since the majority of these injuries have a higher chance of misdiagnosis, we recommend careful clinical examination, a high index of suspicion in cases of high‐energy injury, and taking all three standard view radiographs of the hand.

**Summary**

- Carpometacarpal joints injuries are mostly misdiagnosed, its recommended to obtain all standard radiography of hand including true lateral view.

## 1 Introduction

Finger carpometacarpal fracture‐dislocations are very rare, comprising less than 1% of all hand injuries. More than half of these injuries are commonly misdiagnosed or missed at initial presentation [1].

High levels of suspicion and meticulous clinical examination with proper investigation avoid missing out on such injuries [2].

Early recognition and management have advantages of the possibility of achieving close reduction and percutaneous fixation. If this injury is missed and prolonged for weeks, it requires an open reduction and fixation [2].

Management in an acute setting is mainly close reduction and pinning. In chronic cases and irreducible injuries need to undergo open reduction and fixation. Reduction of these injuries is hampered by interposition of soft tissue and carpal fractures [3].

The purpose of reporting this case is to highlight the importance of detailed examination and a high level of suspicion in cases of high‐energy injuries like motor vehicle accidents. In addition, it emphasizes the importance of taking all three standard view radiographs of thehand.

## 2 Material and Methods

### 2.1 Patient Information

A 31‐year‐old male presented to the orthopedic outpatient department with a 1‐month history of persistent pain and swelling in his right hand following a motor vehicle accident. He had initially received treatment from an orthopedic surgeon elsewhere in Bhutan.

### 2.2 Clinical Features

**FIGURE 1:**
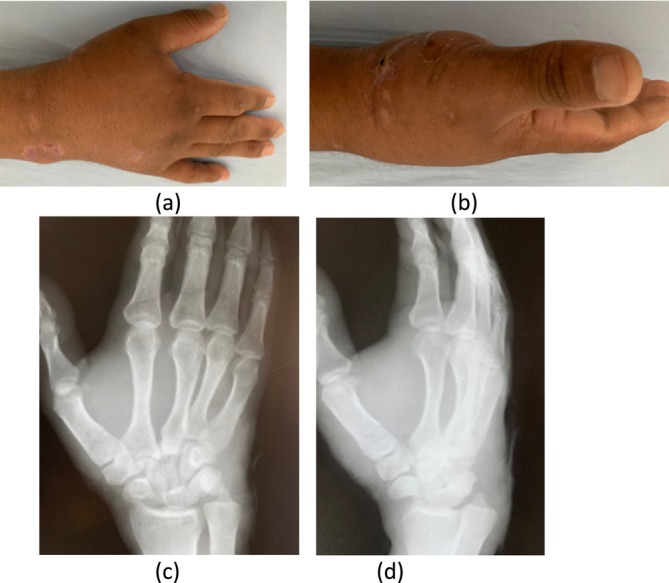
(a) Clinical photo of hand back view, (b) clinical photo side view. Initial *x*‐rays of hand (c) AP view and (d) Oblique view.

On examination, the patient exhibited swelling, deformity, and tenderness over the dorsal aspect of his right hand. He experienced pain while attempting to make a fist, scoring 5 on the Visual Analogue Scale (VAS). His grip strength was significantly reduced to 3 kg in the right hand compared to 11 kg in the left hand. The QuickDASH‐9 score was recorded as 26. Clinical photos are shown in Figure 1a–d.

### 2.3 Imaging

**FIGURE 2:**
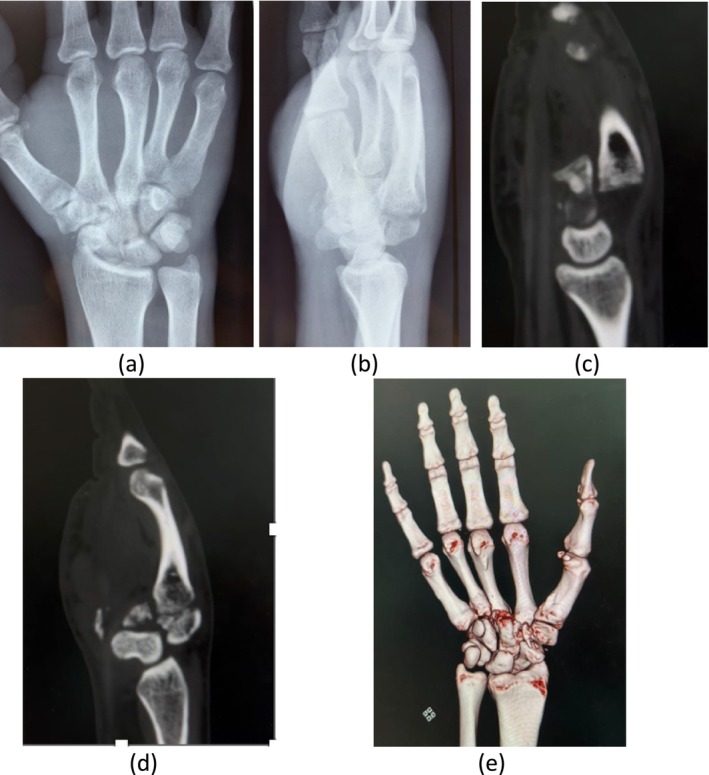
(a) *x*‐ray of hand in AP view, (b) lateral view, (c) CT scan sagittal view of hand showing dorsal displacement of 3rd metacarpal, (d) CT scan sagittal view of hand showing fracture of trapezoid along with dorsal displacement of 2nd metacarpal, (e) CT scan 3D model of hand showing disruption in 2nd and 3rd CMC joint articulation.

Standard anteroposterior (AP) and lateral radiographs of the hand revealed dislocation of the second and third carpometacarpal (CMC) joints (Figure 2a,b). A CT scan of the hand was performed to better define the injury and rule out associated fractures of the metacarpals. The CT scan revealed a comminuted fracture of the trapezoid along with dorsal dislocations of the second and third metacarpals (Figure 2c–e).

### 2.4 Intervention

The procedure was performed under general anesthesia. The second and third CMC joints were approached dorsally between the second and third extensor compartments. Intraoperatively, a comminuted fracture of the trapezoid and dorsal dislocations of the second and third metacarpals were identified.

Reduction could not be achieved with the dorsal approach alone and required releasing the volar capsule using a periosteal elevator. Due to the severity of the trapezoid comminution, reconstruction was not feasible. As a result, the articular cartilage of the base of the second metacarpal was shaved off to allow for future arthrodesis.

**FIGURE 3:**
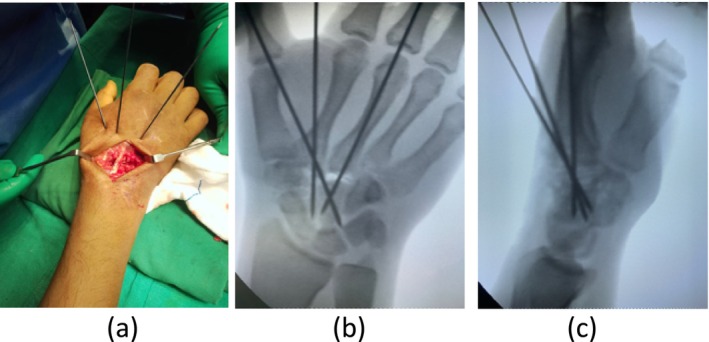
(a) Intra‐operative image showing percutaneous pinning, (b) per‐operative C‐arm images in AP view and (c) lateral view.

The adequacy of reduction was confirmed under fluoroscopy (C‐arm) in Figure 3a–c, and fixation was achieved using K‐wires. One K‐wire was placed from the base of the second metacarpal to the trapezoid, and another from the same origin to the capitate. A third K‐wire was introduced from the base of the third metacarpal to the capitate.

**FIGURE 4:**
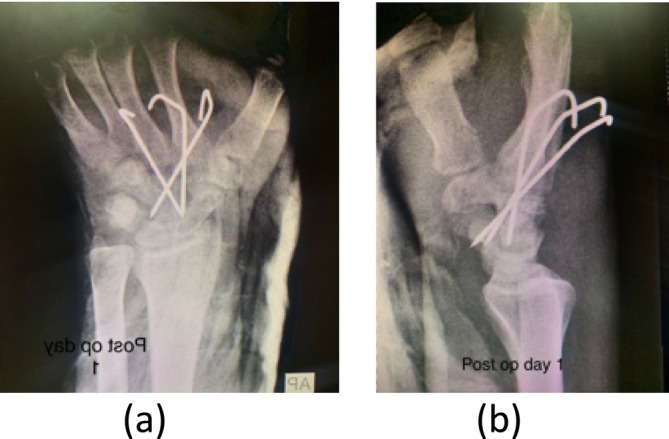
Post operative *x*‐ray with K‐wires in situ (a) AP view and (b) lateral view.

Postoperatively, a short‐arm dorsal splint was applied for 6 weeks. Confirmed K‐wire placement in post‐op *x*‐rays (Figure 4a,b). The K‐wires were removed after 6 weeks, and hand physiotherapy was initiated following their removal.

## 3 Follow‐Up Evaluation

### 3.1 Radiographic Assessment

**FIGURE 5:**
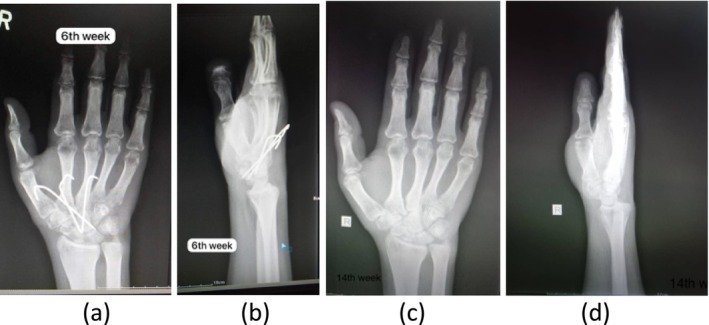
Follow‐up *x‐*rays of hand at 6 weeks (a) AP and (b) lateral views, at 14 weeks (c) AP and (d) lateral views.

- *Six weeks postoperatively*: follow‐up *x*‐rays (Figure 5a,b) showed well‐reduced joints in both AP and lateral views.
- *Fourteen weeks postoperatively*: radiographs (Figure 5c,d) confirmed continued joint stability and proper healing.

### 3.2 Clinical Outcomes

**FIGURE 6:**
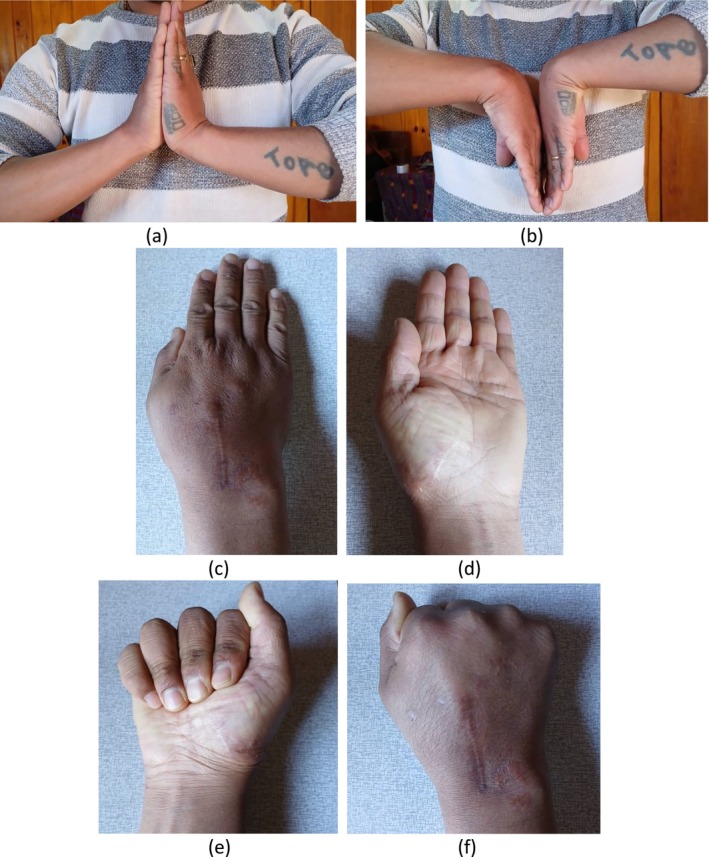
Shows clinical photos after 1 year post operation. Showing full range of wrist motion (a, b). Full finger movements as depicted in image (c–f).

- Three‐month follow‐up:
  ○ *Pain assessment*: VAS score improved to 2.
  ○ *Grip strength*: increased to 10 kg.
  ○ *Functional outcome*: QuickDASH‐9 score improved to 13.
- One‐year postoperative follow‐up (Figure 6):
  ○ *Wrist motion*: full range of motion demonstrated (Figure 6a,b).
  ○ *Finger movements*: normal and unrestricted finger movements were observed (Figure 6c–f).

## 4 Discussion

Finger CMC joint stability is provided by four systems of ligaments. The V‐configuration of the interosseous ligament is one of the strongest among the four ligaments [4].

In addition to a strong ligament configuration, the index finger metacarpal has a wedge‐shaped articulation with the trapezoid making the joint more stable compared to other finger CMC joints [5].

Hence, fracture‐dislocation among finger CMC, especially the 2nd CMC joint, is very rare [6] but unfortunately, the majority, up to 70%, of these injuries are missed [7].

Radiographic features of the carpometacarpal joint seem so simple when you look at first glance. But it is very likely to miss these injuries and very important to include all standard views of the hand, AP, lateral, and oblique views [4]. Other radiological views that will help in diagnosis are parallelisms in the second row of the carpus by fisher et al. [8] and lines of the metacarpal cascade on the PA view [9].

In this case, the injury had been missed because the treating surgeon had done only two‐view radiographs of the hand. The true lateral view of the metacarpal, which is a very important view that helps in diagnosing these injuries, was not obtained.

Early recognition of this injury is critical for management [4]. If the injury is acute, closed reduction is possible and can be managed with closed reduction and percutaneous pinning. However, in cases of late injury, closed reduction becomes difficult and mostly, they need open reduction. In some cases, even if the injury is acute, closed reduction may not be possible or adequate due to interposition of soft tissue and associated fractures; in such cases, also open reduction and fixation are required similar to chronic injuries [3]. At the 1‐year follow‐up, the patient reported no pain and demonstrated a full range of motion in the fingers and wrist joint, performing all activities without any limitations, as shown in Figure 6.

## 5 Conclusion

Uncommon injuries to the finger CMC joints are often overlooked. High‐energy trauma to the hands, such as that sustained in motor vehicle accidents, requires a thorough clinical examination and a high degree of suspicion. Obtaining all standard radiographic views of the hand is essential for identifying these injuries. Acute injuries are generally simpler to manage and require less extensive treatment compared to chronic fracture–dislocations.

## 6 Strengths of the Study

This study highlights what was overlooked during the initial evaluation, such as the failure to obtain a true lateral view of the hand. In the future, treating surgeons will be encouraged to focus on obtaining all standard radiographic views of the hand.

## 7 Limitations of the Study

This case is self‐reported, which introduces the possibility of bias in quantifying the clinical outcome findings. Additionally, as this is a single case, the results cannot be generalized to a larger population.

## Author Contributions

**Yeshi Dorji:** conceptualization, investigation, methodology, validation, writing – original draft, writing – review and editing. **Ugyen Thrinley:** supervision, validation, writing – review and editing.

## Disclosure

The authors declare that there is no relevant or material financial interest that relates to this case report.

## Consent

Written informed consent was taken from the patient.

## Conflicts of Interest

The authors declare no conflicts of interest.

## Data Availability

Data sharing not applicable as no data is available regard to this case report.
